# A multi-level attention CNN-transformer based framework for the detection of brain tumor using regional dual-score explainability

**DOI:** 10.1038/s41598-026-52658-6

**Published:** 2026-05-21

**Authors:** Aryaman Kaprekar, Anjan Gudigar, U. Raghavendra, Mahesh Anil Inamdar, Massimo Salvi, Priyanka, U. R. Acharya

**Affiliations:** 1 Manipal Institute of Technology, Manipal Academy of Higher Education, Manipal, 576104 India; 2 Biolab, PolitoBIOMed Lab, Department of Electronics and Telecommunications, Politecnico di Torino, Corso Duca degli Abruzzi 24, Turin, 10129 Italy; 3 Department of Medical Imaging Technology, Manipal College of Health Professions, Manipal Academy of Higher Education, Udupi, Manipal India; 4 School of Mathematics, Physics, and Computing, University of Southern Queensland, Springfield, QLD 4300 Australia; 5 Centre for Health Research, University of Southern Queensland, Springfield, Australia

**Keywords:** Brain tumor classification, Magnetic Resonance Imaging, Hybrid deep learning, Explainable artificial intelligence, Medical image analysis, Cancer, Computational biology and bioinformatics, Health care, Mathematics and computing, Medical research, Oncology

## Abstract

Deep learning models for brain tumor diagnosis often lack interpretability beyond qualitative visual heatmaps. Clinicians require not only tumor localization but also quantitative assessment of explanation quality and diagnostic relevance, capabilities absent in conventional explainability methods. This paper introduces a classification-explainability framework addressing these limitations. The Multi-Level Hybrid Network (MLHnet) integrates CNN-Transformer components with multi-level attention for robust feature learning. The key novelty lies in the Dual-Score Regional XAI framework, which: (1) identifies tumor regions using hybrid saliency maps combining gradient-based and activation-based information; (2) quantifies geometric tumor characteristics via a Shape Score capturing size, circularity, and saliency concentration; and (3) evaluates explanation faithfulness using regional perturbation analysis, measuring prediction sensitivity to tumor-specific versus non-tumor regions. The framework is evaluated on a publicly available brain Magnetic Resonance Imaging (MRI) dataset containing 7,023 images across four classes. Using five‑fold cross‑validation, the proposed model achieves an average test accuracy of 99.30% (best fold: 99.47%). Expert radiologist evaluation confirms 87.64% explanation correctness, with the Dual-Score metrics successfully differentiating tumor classes based on morphological and saliency patterns. Overall, the proposed framework offers a lightweight, high-performing, and interpretable solution for reliable brain tumor diagnosis from MRI scans on the evaluated public dataset.

## Introduction

Brain tumors are one of the most life-threatening neurological abnormalities, often leading to critical conditions if not diagnosed and treated at an early stage^1,2^. Magnetic Resonance Imaging (MRI) is the most widely used non-invasive imaging modality for brain tumor diagnosis. Manual interpretation of MRI scans is time-consuming, cognitively demanding, and prone to human error.

Artificial Intelligence-based medical image analysis has gained significant attention over the past few decades. Traditional Machine Learning (ML) approaches rely on prepared features and classical classifiers, which often struggle to achieve high accuracy^3^. In the past decade, with the easy availability of sophisticated computational resources, Deep Learning (DL) techniques have displayed better performance by learning hierarchical features directly from image data. Pretrained Convolutional Neural Networks (CNNs) can be fine-tuned and used to perform brain tumor-related tasks such as classification and segmentation accurately^4^. Vision Transformers (ViT)^5^ have been explored in recent years for medical image diagnosis due to their attention-based representations^6^. These advances highlight the potential of DL to serve as reliable support tools for radiologists, with the capability to identify complex patterns, and overcome human bias and error. However, effectively capturing tumor characteristics across multiple spatial scales while maintaining robust generalisation remains a challenging problem.

Despite their impressive performance, DL models retain a black-box nature, posing critical challenges in deployment for clinical practice, where transparency, trust and accountability are essential^7^. Explainable Artificial Intelligence (XAI)^8^ addresses these concerns by providing interpretable explanations to model predictions. In image classification, XAI techniques such as Gradient-weighted Class Activation Mapping (Grad-CAM)^9^, Occlusion^10^ and Local Interpretable Model-agnostic Explanations (LIME)^11^ have been widely adopted to highlight salient regions of the input. Using such methods, practitioners can verify if the model predicts based on meaningful tumor regions rather than random correlations. Such expert-AI collaboration can make medical diagnosis faster, more reliable and less laborious while improving accuracy, maintaining trust and transparency. However, most existing approaches provide pixel‑level explanations and lack mechanisms to assess whether the highlighted regions correspond to clinically meaningful abnormalities.

In this study, we propose a unified deep learning framework that integrates brain tumor classification with abnormal region localization and explainability. We compare the proposed methodology with various state-of-the-art models and XAI methods. The contribution of this study is as follows:

- Multi-Level Hybrid Network (MLHnet), a CNN-Transformer hybrid that leverages mid-level and final-level feature representations to capture tumor texture and high-level semantic information.
- Refinement of multi-level features using feature enhancing modules like Convolution Block Attention Module (CBAM)^12^ and Generalised Mean (GeM) Pooling^13^. These components improve feature discrimination and robustness.
- Dual-Score Regional XAI, which uses hybrid saliency to localize abnormal tumor regions and evaluates explanation faithfulness, through a perturbation-based test.

The remainder of the paper is organized into six sections. Section  2 lists and reviews current research and state-of-the-art literature on brain tumor classification and XAI. Section  3 explains the architecture and process of the proposed methodology. Section  4 presents the experimentation setup, results and the ablation study. Section  5 is the discussion where we explain and analyse the results, compare the proposed method with current state-of-the-art studies and explore the limitations and future directions. Section  6 summarises and concludes the proposed research.

## Literature review

Brain tumor classification from MRI scans has seen rapid development with the advent of deep learning, particularly convolutional neural networks (CNNs). Early CNN-based approaches focused on local regions, textures and edges, with studies showing that tumor‑focused data augmentation and deeper architectures with small convolutional kernels improved classification performance^14^. As CNNs became dominant, transfer learning gained popularity such as^15^ where Deepak & Ameer fine-tuned pretrained CNNs with standard classifiers and reported strong multi-class performance for that time. Afshar et al.^16^ proposed a modified CapsNet which used coarse tumor boundary information as an input to reduce sensitivity to background and improve brain tumor classification performance. Beyond CNNs, DDFC^17^ proposes an attention-integrated architecture based on Gated Recurrent Units (GRUs), a lightweight recurrent neural network variant designed to model sequential dependencies through update and reset gates, in order to enhance feature selectivity. Brain Tumor Detection Network (BTDN)^18^ proposes a secure data transmission method for MRI-based diagnosis while achieving highly accurate classification performance. Despite their strong performance, CNN-based models primarily learn local texture, edge, and morphological patterns, and may therefore have limited ability to capture long-range contextual dependencies that can also be important for distinguishing tumor subtypes.

Dosovitskiy et al.^5^ introduced vision transformers, demonstrating that a patch-based self-attention can be effective for image recognition. In medical imaging, a comparative study^19^ evaluated multiple ViT variants for brain tumor image classification. It reported strong results demonstrating that ViTs are effective backbones when pretrained weights are available. Hierarchical transformer designs such as Swin Transformer^20^ restrict attention to shifted local windows while still allowing cross window interactions, also introducing relative position bias. Several segmentation‑assisted pipelines have been explored to localize tumor regions prior to classification, including transformer‑based segmentation models^21^, object‑detection‑driven approaches^22^, and explainability‑oriented segmentation frameworks such as Neuro‑XAI^23^. While Transformer-based methods improve global context modelling through self-attention, they typically involve higher computational complexity and often benefit from large-scale pretraining, which may limit their practicality in data-constrained or resource-constrained clinical settings.

Hybrid architectures combine CNNs’ local features with Transformers’ global context. For example, CAFNet^24^ integrates a CNN and a ViT via a Cross-Attention Fusion (CAF) module. Napa et al.^25^ propose BRAIN-META, an ensemble of ten hybrid CNN–ViT models. Each base model fuses a pretrained CNN with a ViT block then stacks the softmax outputs into meta-features, and trains an XGBoost meta-learner. Swaminathan et al.^26^ present a DenseNet and Custom-CNN hybrid, a DenseNet121 backbone concatenated with a tailor-made CNN head for binary tumor detection. EFFResNet-ViT^27^ is a fusion approach that combines EfficientNetB0 and Resnet50 to extract features, followed by a ViT module, which yields a high accuracy of 99.31% in multiclass brain tumor classification. However, many existing hybrid methods rely on single-level or late-stage feature aggregation and do not explicitly exploit the complementary information contained in intermediate and high-level representations through dedicated multi-level fusion and refinement mechanisms.

Recent Deep Learning research has integrated XAI in its workflow. GradCAM heatmaps, used to explain image regions driving predictions, are the most commonly used XAI method^25,28,23,29,18,27^. SHAP (SHapley Additive exPlanations)^30^, based on cooperative game theory, is another popular XAI method used by^31 and 32^.

Current XAI methods highlight tumor-relevant regions; however, they do not adequately explain why the model predicts a specific class based on those regions. Moreover, most existing approaches lack quantitative measures to assess the faithfulness of the highlighted regions. These gaps motivate the proposed approach, in which MLHnet focuses on holistic learning from brain tumor scans and the Dual-Score Regional XAI demonstrates both model-agnostic and class-agnostic behavior.

## Proposed methodology

**Fig. 1:**
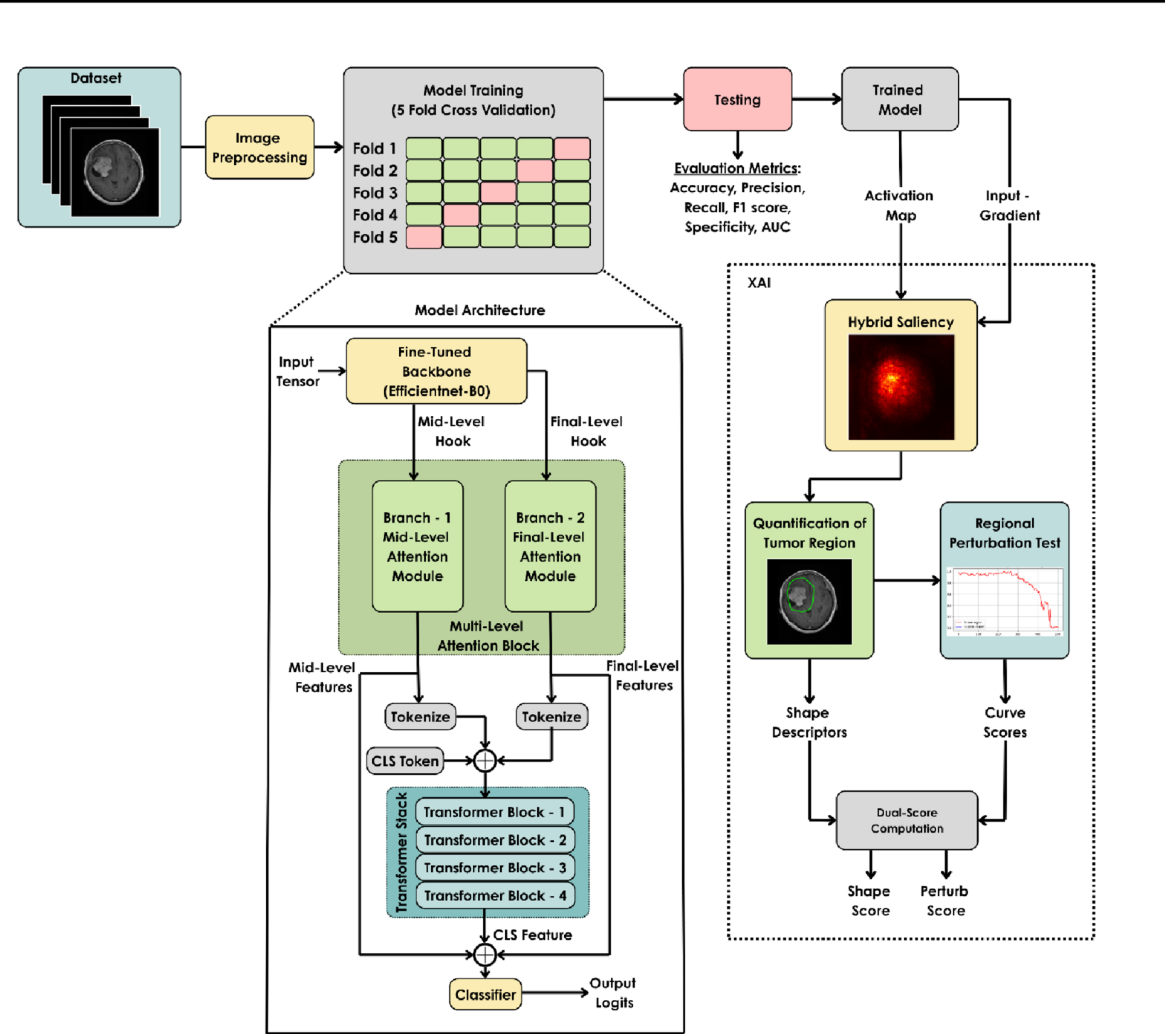
Overview of the proposed methodology. Brain MRI images are preprocessed and used to train the MLHnet model through 5-fold cross-validation. The best-performing model is selected based on validation metrics and subsequently employed for Dual-Score Regional XAI analysis on the test set.

The proposed model is a unified framework for classification and explainability. The MLHnet serves as the classification module and is based on a Hybrid CNN-Transformer architecture. It incorporates a Multi-Level Attention Block (MAB) and a fusion classifier head to capture discriminative features. Predictions made by MLHnet are then explained using the Dual Score Regional XAI module, which uses hybrid saliency maps to localize tumor regions. The faithfulness of the localized regions is evaluated through a regional perturbation test. The complete model architecture and workflow are illustrated in Fig. 1.

### Multi-level hybrid network (MLHnet)

CNN-Transformer hybrid models have gained attention in medical image analysis for their ability to combine local feature extraction with contextual representation. In brain tumor diagnosis, both regional feature discrimination and accurate localisation of abnormalities are required, often under limited positive samples, making hybrid representations particularly effective.

This paper proposes MLHnet, a CNN-Transformer hybrid that utilises multi-level attention modules to enhance feature representation across classes. A CNN backbone extracts features at intermediate and final stages which are processed in the Multi-level Attention Block and passed to a 4-block transformer. The classifier then concatenates all extracted features and outputs the prediction logits.

#### Backbone- EfficientNet-B0

The CNN backbone used for feature extraction is the EfficientNet-B0^33^. EfficientNet-B0 is the baseline EfficientNet model proposed by Tan and Le. It achieves high accuracy with very low memory requirements and computational costs. It uses Mobile Inverted Bottleneck Convolutions (MBConv), Squeeze and Excitation (SE) modules, depth-wise separable convolutions and Swish activation functions. With only 5.3 million parameters and 7 blocks, EffecientNet-B0 was selected as the backbone feature extractor. This choice was motivated by its favourable balance between representational capacity and computational efficiency. This is particularly suitable for medical imaging settings where lightweight models with strong discriminative performance are desirable.

We input an image denoted as $\:\mathbf{I}\in\:{\mathbb{R}}^{H\times\:W\times\:C}$ to a sequence of EfficientNet-B0 blocks where each block produces feature maps passed on to the next block:

1 $$\:\begin{array}{cccc}&\:{\mathbf{F}}_{l}={\mathcal{E}}_{l}\left(\mathbf{I}\right),l=1,\dots\:,7\:&\:&\:\end{array}$$

where $\:{\mathcal{E}}_{l}(\cdot\:)$represents the transformation performed by the *\:l*-th EfficientNet block; H, W and C are the height, width and channel (3) of the input image. Instead of using only the final level to extract features, we additionally extract features from an intermediate stage in order to combine complementary representations at different semantic levels. Intermediate features preserve richer spatial and texture-related information, while deeper features capture more abstract semantic patterns that are useful for tumor subtype discrimination. Based on this rationale, two feature maps of different resolutions are extracted: one from the 4th block ($\:{\mathbf{F}}_{\mathrm{mid}})$ and one from the 7th block $\:\left({\mathbf{F}}_{\mathrm{final}}\right).$ The 4th block was selected as a representative intermediate stage with sufficient spatial detail, while the 7th block provides the final high-level semantic representation.

The mid-level feature map is extracted after the first four stages of EfficientNet-B0, corresponding to:

2 $$\:\begin{array}{cccc}&\:{\mathrm{F}}_{\mathrm{mid}}={\mathcal{E}}_{1:4}\left(\mathrm{I}\right),&\:&\:\end{array}$$

This feature map has: $\:{\mathrm{F}}_{\mathrm{mid}}\in\:{\mathbb{R}}^{{C}_{m}\times\:{H}_{m}\times\:{W}_{m}},$ where the channel dimension $\:{C}_{m}=80$.

The final-level feature map is extracted after all seven stages of EfficientNet-B0, corresponding to:

3 $$\:\begin{array}{cccc}&\:{\mathrm{F}}_{\mathrm{final}}={\mathcal{E}}_{1:7}\left(\mathrm{I}\right),&\:&\:\end{array}$$

This feature map has: $\:{\mathrm{F}}_{\mathrm{final}}\in\:{\mathbb{R}}^{{C}_{f}\times\:{H}_{f}\times\:{W}_{f}},$ where the channel dimension $\:{C}_{f}=1280$.

#### Multilevel Attention Block (MAB)

**Fig. 2:**
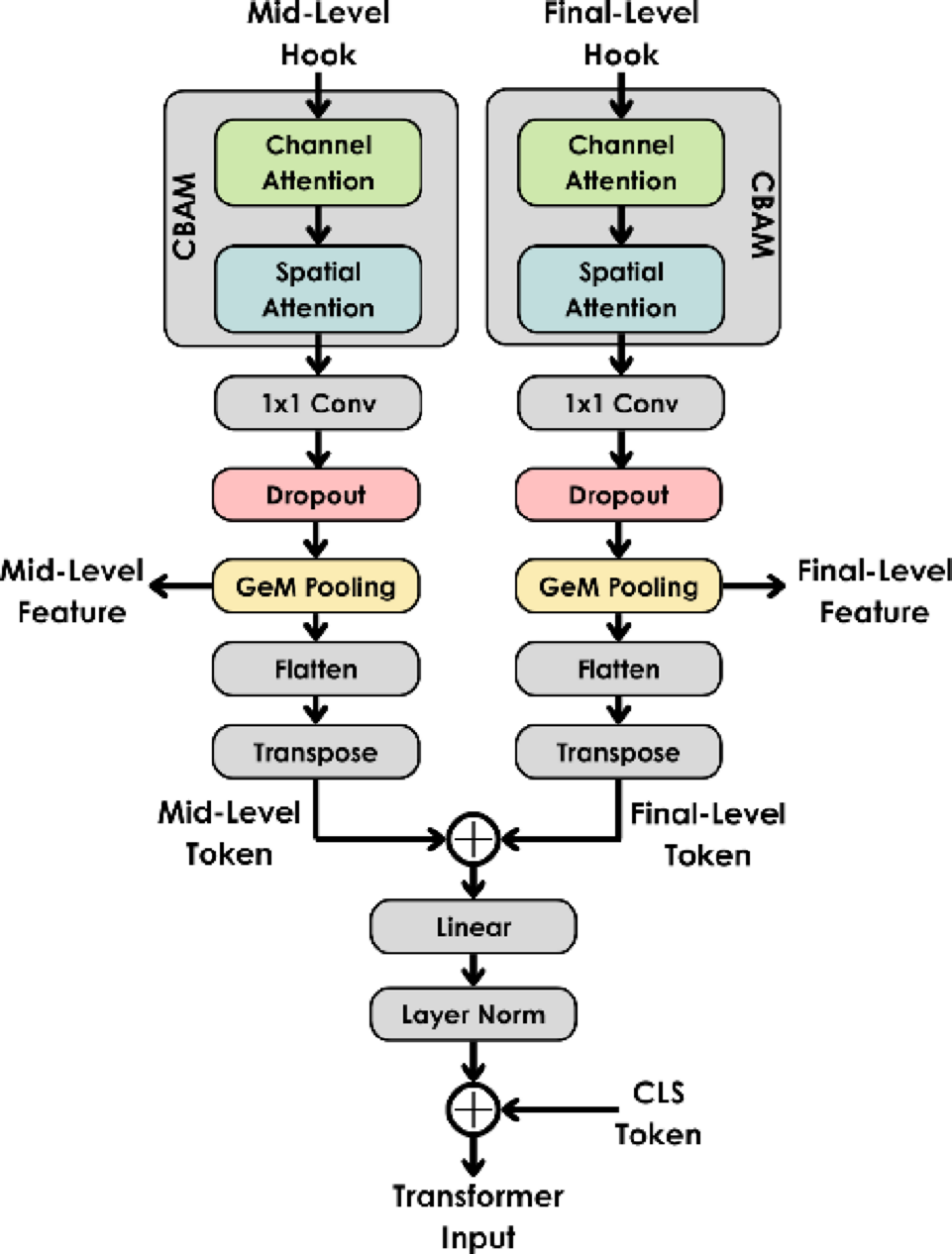
Architecture of the MAB. The block consists of two parallel branches that process mid-level and final-level features. These features are then tokenized and concatenated with a learnable class token before being fed to the transformer encoder.

The MAB consists of two branches: one for the intermediate level and the other for the final level (see Fig. 2). Each MAB has a Convolutional Block Attention Module (CBAM), which sequentially applies channel attention followed by spatial attention.

Given an input feature map $\:\mathbf{F}\in\:{\mathbb{R}}^{C\times\:H\times\:W}$, channel attention aggregates global spatial information using both average pooling and max pooling:

4 $$\:\begin{array}{cccc}&\:{\mathbf{z}}_{\mathrm{avg}}=\mathrm{G}\mathrm{A}\mathrm{P}\left(\mathbf{F}\right),{\:\mathbf{z}}_{\mathrm{max}}=\mathrm{G}\mathrm{M}\mathrm{P}\left(\mathbf{F}\right)&\:&\:\end{array}$$

Where GAP stands for Global Average Pooling and GMP stands for Global Max Pooling.

These are passed to a Multi-Layer Perceptron (MLP) with sigmoid function ($\:\sigma\:)$ to generate channel attention weights:

5 $$\:\begin{array}{cccc}&\:{\mathbf{M}}_{c}=\sigma\:\left(\mathrm{M}\mathrm{L}\mathrm{P}\left({\mathbf{z}}_{\mathrm{avg}}\right)+\mathrm{M}\mathrm{L}\mathrm{P}\left({\mathbf{z}}_{\mathrm{max}}\right)\right)&\:&\:\end{array}$$

The feature is refined via channel-wise multiplication:

6 $$\:\begin{array}{cccc}&\:{\boldsymbol{F}}_{\boldsymbol{c}}={\mathbf{M}}_{c}\odot\:\mathbf{F}&\:&\:\end{array}$$

Spatial attention on the contrary, emphasizes salient regions by aggregating channel information. Given$\:{\:\boldsymbol{F}}_{\boldsymbol{c}}$, spatial descriptors are computed as:

7 $$\:\begin{array}{cccc}&\:{\mathbf{s}}_{\mathrm{avg}}={\mathrm{A}\mathrm{v}\mathrm{g}\mathrm{P}\mathrm{o}\mathrm{o}\mathrm{l}}_{\mathrm{channel}}\left({\boldsymbol{F}}_{\boldsymbol{c}}\right),\:{\mathbf{s}}_{\mathrm{max}}={\mathrm{M}\mathrm{a}\mathrm{x}\mathrm{P}\mathrm{o}\mathrm{o}\mathrm{l}}_{\mathrm{channel}}\left({\boldsymbol{F}}_{\boldsymbol{c}}\right)&\:&\:\end{array}$$

These are concatenated and convolved to produce a spatial attention map:

8 $$\:\begin{array}{cccc}&\:{\mathbf{M}}_{s}=\sigma\:\left({f}^{7\times\:7}\left([{\mathbf{s}}_{\mathrm{avg}};{\mathbf{s}}_{\mathrm{max}}]\right)\right)&\:&\:\end{array}$$

where $\:{f}^{7\times\:7}$ is the 2D convolution operation with $\:7\times\:7$ kernel size.

The feature map from CBAM is:

9 $$\:\begin{array}{cccc}&\:{\mathbf{F}}_{\mathrm{att}}={\mathbf{M}}_{s}\odot\:{\boldsymbol{F}}_{\boldsymbol{c}}&\:&\:\end{array}$$

Channel attention focuses on “What features are important?” while Spatial attention answers “Where are the important features located?”.

The two branches of MAB for mid and final level features have different spatial dimensions, so each branch is projected into a common embedding space using 1 × 1 convolution. A dropout rate^34^ of 0.3 is applied after the 1 × 1 convolution layers for regularisation and to prevent overfitting.

10 $$\:\begin{array}{cccc}&\:\widehat{\boldsymbol{F}}=\:{\left({\mathrm{D}\mathrm{r}\mathrm{o}\mathrm{p}\mathrm{o}\mathrm{u}\mathrm{t}}_{0.3}\right(\mathrm{C}\mathrm{o}\mathrm{n}\mathrm{v}}_{1\times1}({\mathbf{F}}_{\mathrm{att}})))&\:&\:\end{array}$$

The GeM pooling is adopted in place of GAP and GMP to reduce over-smoothing and sensitivity to noise. It uses a learnable parameter (p) where *p* = 1 means GeM acts as GAP while p →∞ means GeM acts as GMP. GeM pooling computes for each channel ($\:{\mathbf{g}}_{c})\:$as:

11 $$\:\begin{array}{cccc}&\:{\mathbf{g}}_{c}={\left(\frac{1}{HW}\sum\:_{i=1}^{H}\sum\:_{j=1}^{W}{\left(\mathrm{m}\mathrm{a}\mathrm{x}({\widehat{\boldsymbol{F}}}_{{\:{c,\:i,\:j}}},\epsilon)\right)}^{p}\right)}^{\frac{1}{p}},c=1,\dots\:,d&\:&\:\end{array}$$

Where $\:{\widehat{\boldsymbol{F}}}_{{\:{c,\:i,\:j}}}$ is the refined feature at spatial location {i, j} of the channel c, $\:p\:$ is a learnable pooling parameter and *ε* ensures numerical stability.

This adaptive pooling strategy is beneficial because the resulting representations are used both for token generation in the Transformer module and for the downstream classification stage.

#### Transformer

**Fig. 3:**
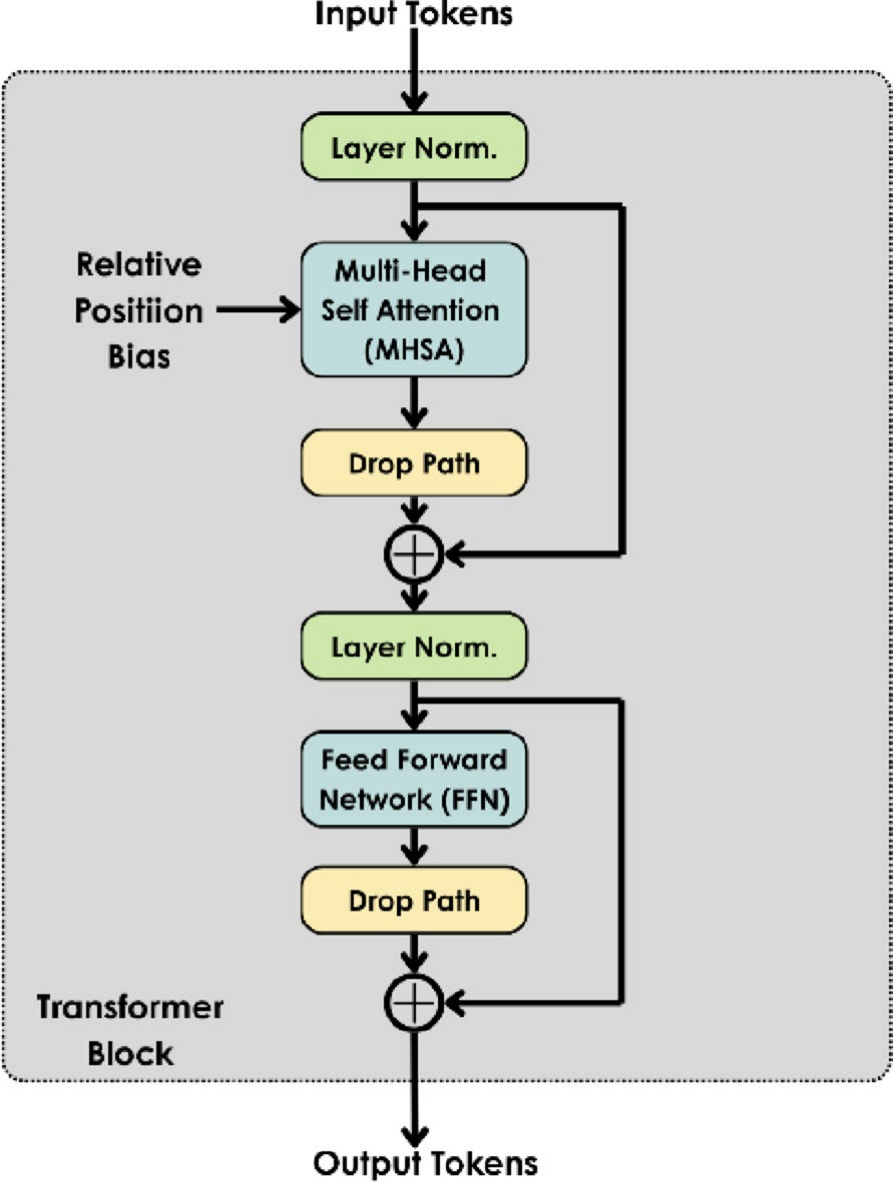
Architecture of a single transformer block. The input token sequence undergoes Multi-Head Self Attention, followed by a Feed-Forward Network.

The transformer enables the model to capture long-range/ global context. It has four blocks stacked sequentially with each block following the architecture illustrated in Fig. 3.

The input received from the MAB is tokenized by flattening, linear projection and normalisation. A learnable class token ($\:{\mathbf{t}}_{\mathrm{cls}}$) is added to form transformer output features.

12 $$\:\begin{array}{cccc}&\:\boldsymbol{Z}=\left[{\mathbf{t}}_{\mathrm{cls}};{\mathbf{T}}_{\mathrm{mid}}\text{};{\mathbf{T}}_{\mathrm{final}}\right]&\:&\:\end{array}$$

where $\:{\mathbf{T}}_{\mathrm{mid}}$ and $\:{\mathbf{T}}_{final}$ are the mid and final level token obtained from tokenization of mid and final features respectively.

These tokens are then sent to the Multi-Head Self-Attention (MHSA) blocks. The Relative Position bias^20^ encodes the relative distance between tokens which preserves spatial context after flattening. It is added to the scaled dot product attention, after which the attention weights are softmax-normalised. Each transformer block includes a Feed-Forward Network (FFN) implemented as a two-layer MLP with Gaussian Error Linear Unit (GELU) activation. Layer Normalization (LN) and residual connections are applied around both the MHSA and FFN modules:

13 $$\:\begin{array}{cccc}&\:{\mathbf{Z}}^{{\prime\:}}=\mathbf{Z}+\mathrm{D}\mathrm{r}\mathrm{o}\mathrm{p}\mathrm{P}\mathrm{a}\mathrm{t}\mathrm{h}\left(\mathrm{M}\mathrm{H}\mathrm{S}\mathrm{A}\right(\mathrm{L}\mathrm{N}\left(\mathbf{Z}\right)\left)\right)&\:&\:\end{array}$$

14 $$\:\begin{array}{cccc}&\:{\mathbf{Z}}_{out}={\mathbf{Z}}^{{\prime\:}}+\mathrm{D}\mathrm{r}\mathrm{o}\mathrm{p}\mathrm{P}\mathrm{a}\mathrm{t}\mathrm{h}\left(\mathrm{F}\mathrm{F}\mathrm{N}\right(\mathrm{L}\mathrm{N}\left({\mathbf{Z}}^{{\prime\:}}\right)\left)\right)&\:&\:\end{array}$$

DropPath (stochastic depth)^35^ of 0.1 is used to randomly drop residual branches, helping in regularisation. Here, $\:{\mathbf{Z}}_{out}\:$ denotes the output obtained from a single block, whereas $\:{Z}_{cls}$ represents the output derived from the complete four-layer stacked architecture.

#### Fusion Classifier

The final prediction is obtained from a fusion classifier, which combines the CNN Encoder mid-level ($\:{\mathbf{g}}_{\mathrm{mid}}$), final-level CNN ($\:{\mathbf{g}}_{\mathrm{final}}$) and transformer ($\:{\mathbf{Z}}_{cls})$ features to represent texture, semantic and contextual information, respectively. These are concatenated to form a unified feature vector.

15 $$\:\begin{array}{cccc}&\:\mathbf{f}=\left[{\mathbf{g}}_{\mathrm{mid}};{\mathbf{g}}_{\mathrm{final}};{\mathbf{Z}}_{cls}\right]\in\:{\mathbb{R}}^{3d}&\:&\:\end{array}$$

The Classifier Head consists of linear layers for dimensionality reduction, ReLU activations for non-linearity and dropout for regularisation.

16 $$\:\begin{array}{cccc}&\:\widehat{\mathbf{y}}={\mathrm{F}\mathrm{C}}_{2}\left(\mathrm{D}\mathrm{r}\mathrm{o}\mathrm{p}\mathrm{o}\mathrm{u}\mathrm{t}\left(\mathrm{R}\mathrm{e}\mathrm{L}\mathrm{U}\left({\mathrm{F}\mathrm{C}}_{1}\left(\mathbf{f}\right)\right)\right)\right)&\:&\:\end{array}$$

where $\:\widehat{\mathbf{y}}\in\:{\mathbb{R}}^{C}$ denotes the predicted class logits, FC denotes fully connected dense neural network and $\:C\:$ is the number of target classes.

### Dual-Score Regional XAI

**Fig. 4:**
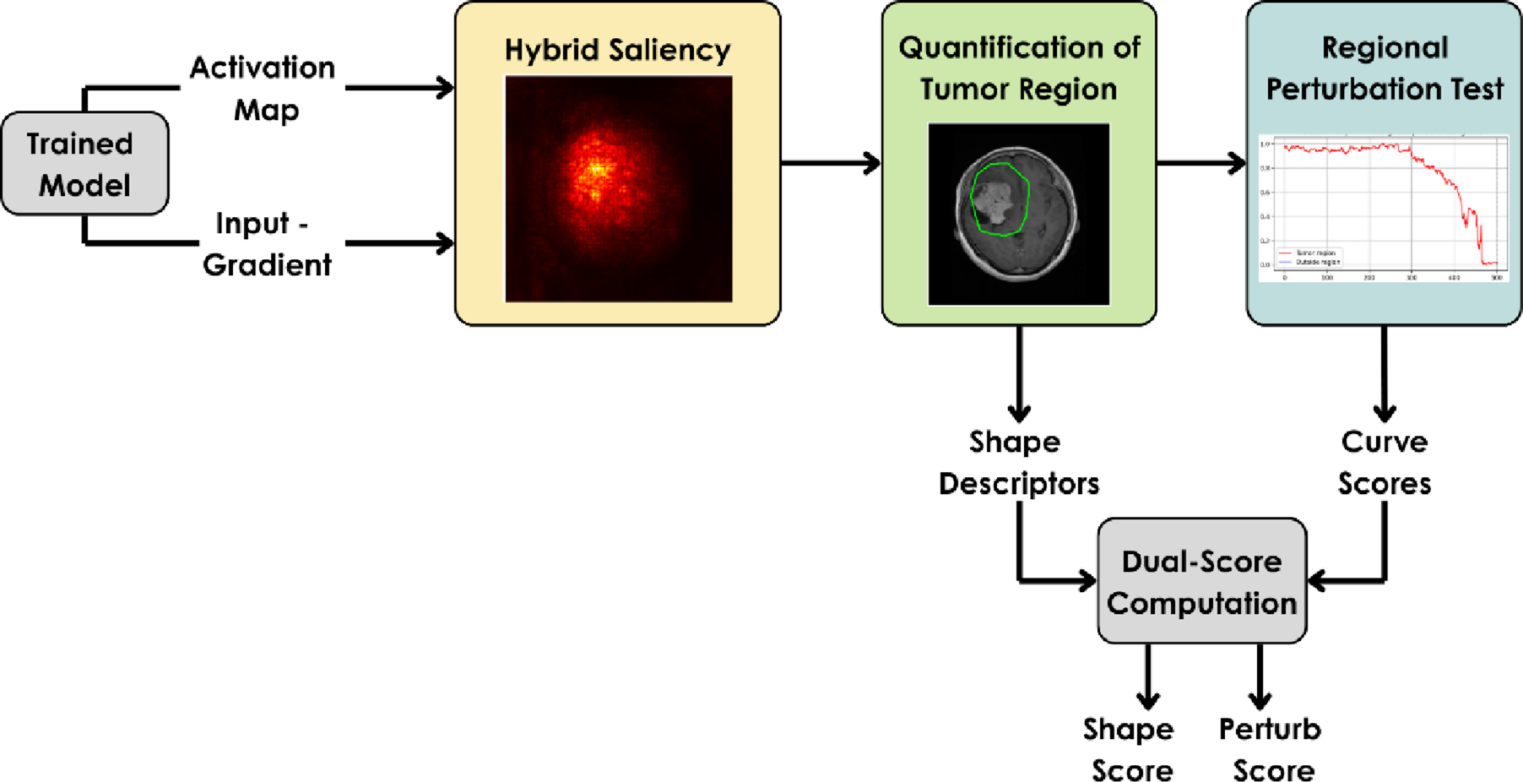
Proposed XAI workflow: Input Gradient and Activation map from the CBAM of the final level attention block are collected from the trained model and fed to the proposed framework.

The Dual-Score Regional XAI framework provides a post-hoc interpretability mechanism for predictions made by the MLHnet. Our method explains the model output after prediction through region-level analysis targeting clinically relevant abnormality regions. A tumor region is defined using hybrid saliency, followed by a Most Relevant First (MoRF)-like perturbation test^36^. The result is a class-agnostic shape score and a perturb score to quantify faithfulness. The proposed XAI workflow is illustrated in Fig. 4.

#### Hybrid Saliency

The hybrid saliency map is generated by combining pixel-level saliency with high-level semantic maps, providing a more stable and robust representation. An input image (**I**) is passed through the trained model. Pixel-level sensitivity $\:{\mathbf{S}}_{\mathrm{grad}}$ (pixel wise gradient saliency map) is computed by backpropagation of the prediction corresponding to the target class.

17 $$\:\begin{array}{cccc}&\:{\mathbf{S}}_{\mathrm{grad}}=\underset{k\in\:\{\mathrm{1,2},3\}}{\mathrm{m}\mathrm{a}\mathrm{x}}\mid\:\frac{\partial\:{f}_{c}\left(\mathbf{I}\right)}{\partial\:{\mathbf{I}}_{k}}\mid\:&\:&\:\end{array}$$

where $\:{f}_{c}$ is the trained model outputs (logits) and $\:k\:$ are the RGB channels. Activation based saliency maps ($\:{\mathbf{S}}_{\mathrm{cam}})$ is hooked from the second branch of the MAB containing the CBAM refined attention map.

18 $$\:\begin{array}{cccc}&\:{\mathbf{S}}_{\mathrm{cam}}=\frac{1}{C}\sum\:_{c=1}^{C}{\boldsymbol{F}}_{\boldsymbol{a}\boldsymbol{t}\boldsymbol{t}}&\:&\:\end{array}$$

The map is up-sampled to the input size using bilinear interpolation. Both maps are min-max normalised to [0,1]. The final hybrid saliency map ($\:{\mathbf{S}}_{\mathrm{hybrid}}$) is computed using elementwise multiplication, which enhances regions that are important across both saliency maps:

19 $$\:\begin{array}{cccc}&\:{\mathbf{S}}_{\mathrm{hybrid}}={\mathbf{S}}_{\mathrm{grad}}^{(1-\alpha\:)}\odot\:{\mathbf{S}}_{\mathrm{cam}}^{\alpha\:}&\:&\:\end{array}$$

where $\:\alpha\:\in\:\left[\mathrm{0,1}\right]$ controls the effect of each saliency map. The fused map is normalised again to maintain consistency in the quantitative range.

#### Quantification of Tumor Region

Rather than relying on predefined manual annotations, the tumor region is inferred from the hybrid saliency map. A binary mask is constructed by applying a high-percentile threshold:

20 $$\:\begin{array}{cccc}&\:\mathbf{M}(x,y)=\left\{\begin{array}{cc}1,&\:{\mathbf{S}}_{\mathrm{hybrid}}(x,y)>{\tau\:}_{p}\\\: 0,&\:\mathrm{otherwise}\end{array}\right.&\:&\:\end{array}$$

where $\:{\tau\:}_{p}$ corresponds to the *\:p*-th percentile. Morphological operation i.e., opening is applied on the binary mask to suppress noisy artifacts. Using connected component analysis, the largest connected region is selected as the abnormality region. Convex hull reconstruction and morphological closing are used for visualising a smoothened circular region. Several shape descriptors are calculated from the detected tumor region such as:

21 $${\rm Area:}\: A=\sum\:_{\left(x,y\right)\in\:{\Omega\:}}{\mathbf{R}}_{\mathrm{tumor}}(x,y)$$

where $\:{\mathbf{R}}_{\mathrm{tumor}}$ represent pixels pertaining to the detected tumor region, and x, y are the pixel coordinates.

22 $${\rm Normalized \:area}:\:{A}_{\mathrm{norm}}=\frac{A}{\mid\:{\Omega\:}\mid\:}\:$$

where $\:\mid\:{\Omega\:}\mid\:$denotes the total number of image pixels.

Saliency-weighted area:

23 $$\:{A}_{\mathrm{weighted}}=\sum\:_{(x,y)\in\:{\Omega\:}}{R}_{\mathrm{tumor}}(x,y){S}_{\mathrm{hybrid}}(x,y)$$

Saliency-weighted Centroid:

24 $$\:{x}_{c}=\frac{\sum\:_{(x,y)\in\:{\mathbf{R}}_{\mathrm{tumor}}}x\cdot\:{\mathbf{S}}_{\mathrm{hybrid}}(x,y)}{\sum\:_{(x,y)\in\:{\mathbf{R}}_{\mathrm{tumor}}}{\mathbf{S}}_{\mathrm{hybrid}}(x,y)}$$

$$\:{y}_{c}=\frac{\sum\:_{(x,y)\in\:{\mathbf{R}}_{\mathrm{tumor}}}y\cdot\:{\mathbf{S}}_{\mathrm{hybrid}}(x,y)}{\sum\:_{(x,y)\in\:{\mathbf{R}}_{\mathrm{tumor}}}{\mathbf{S}}_{\mathrm{hybrid}}(x,y)}$$

Mean Saliency:

25 $$\:{S}_{\mathrm{mean}}=\frac{1}{A}\sum\:_{(x,y)\in\:{\mathbf{R}}_{\mathrm{tumor}}}{\mathbf{S}}_{\mathrm{hybrid}}(x,y)$$

Maximum Saliency:

26 $$\:{S}_{\mathrm{max}}=\underset{(x,y)\in\:{R}_{\mathrm{tumor}}}{\mathrm{m}\mathrm{a}\mathrm{x}}{S}_{\mathrm{hybrid}}(x,y)$$

Normalized Saliency-weighted Area:

27 $$\:{A}_{\mathrm{weighted}}^{\mathrm{norm}}=\frac{{A}_{\mathrm{weighted}}}{\sum\:_{\left(x,y\right)\in\:{\Omega\:}}{\mathbf{S}}_{\mathrm{hybrid}}\left(x,y\right)}$$

28 $${\rm Circularity:\hat{C}=\frac{4\pi\:A}{P^{2}}}$$

where *\:P* denotes the perimeter of $\:{\mathbf{R}}_{\mathrm{tumor}}$.

#### Regional Perturbation Test

To evaluate the faithfulness of the generated explanations, we perform a MoRF-like region-aware Perturbation test. This test evaluates if the salient pixels, especially those within the computed region, are influential to the model output. Given the saliency map $\:{\mathbf{S}}_{\mathrm{hybrid}}$, all pixels are ranked in descending order of saliency. The top $\:K\mathrm{\%}\:$ most salient pixels are selected for perturbation. The selected top-*\:K* pixels are divided into two groups:

1. Pixels inside the tumor region: Pixels lying within the boundary of the generated tumor region.
2. Pixels outside the tumor region: Pixels lying outside the generated tumor region, within and outside the brain.

Perturbations are applied sequentially, first to tumor-region pixels and then to outside pixels. This allows the demonstration of faithfulness of the tumor region. Each pixel is then perturbed by replacing its value with a value corresponding to the dataset mean (µ). After each step, the predicted score for the target class is recorded. The resulting score sequence is normalized to the range $\:\left[0,1\right]$ to enable comparison across samples.

#### Dual-score computation

The framework uses two scores to quantify the XAI representation. The shape-based score ($\:{T}_{\mathrm{shape}}$) captures localized spatial characteristics in a class-agnostic manner, yielding different value ranges for different classes under the same scoring logic. This enables to quantify how the model differentiates between classes based on geometry. The perturbation-based score ($\:{T}_{\mathrm{perturb}}$) helps quantify the faithfulness of the model explanations.

The shape score is computed as a weighted combination of metrics derived from the quantification of the tumor region. It is defined as:

29 $$\:{T}_{\mathrm{shape}}={w}_{1}\widehat{C}+{w}_{2}{S}_{\mathrm{mean}}+{w}_{3}{A}_{\mathrm{weighted}}^{\mathrm{norm}}+{w}_{4}{A}_{\mathrm{norm}}$$

where $\:\:\left\{{w}_{i}\right\}\:$ are empirically determined weights satisfying $\:{\sum\:}_{i}{w}_{i}=1$. The resulting score is clipped to the range [0,1].

From the regional perturbation test, we obtain two confidence curves:

- $\:{\mathbf{C}}_{\mathrm{tumor}}$: scores after perturbing tumor-region pixels,
- $\:{\mathbf{C}}_{\mathrm{outside}}$: scores after perturbing non-tumor pixels.

The Area Under Curve (AUC) is computed and normalized:

30 $$\:{\mathrm{Drop}}_{\mathrm{tumor}}=1-\mathrm{AUC}\left({\mathbf{C}}_{\mathrm{tumor}}\right)$$

31 $$\:{\mathrm{Drop}}_{\mathrm{outside}}=1-\mathrm{AUC}\left({\mathbf{C}}_{\mathrm{outside}}\right)$$

A separation term is used to emphasize the tumor region. It is defined as:

32 $$\:\mathrm{Separation}=\frac{{\mathrm{Drop}}_{\mathrm{tumor}}}{{\mathrm{Drop}}_{\mathrm{tumor}}+{\mathrm{Drop}}_{\mathrm{outside}}}$$

The perturbation-based trust score is then computed as:

33 $$\:{T}_{\mathrm{perturb}}=b\cdot\:{\mathrm{Drop}}_{\mathrm{tumor}}+(1-b).\mathrm{S}\mathrm{e}\mathrm{p}\mathrm{a}\mathrm{r}\mathrm{a}\mathrm{t}\mathrm{i}\mathrm{o}\mathrm{n}$$

where *b **∈*[0,1] controls the effect of Tumor Drop on the perturb score.

The complete Dual-Score Regional XAI algorithm is given below:

**Figure:**
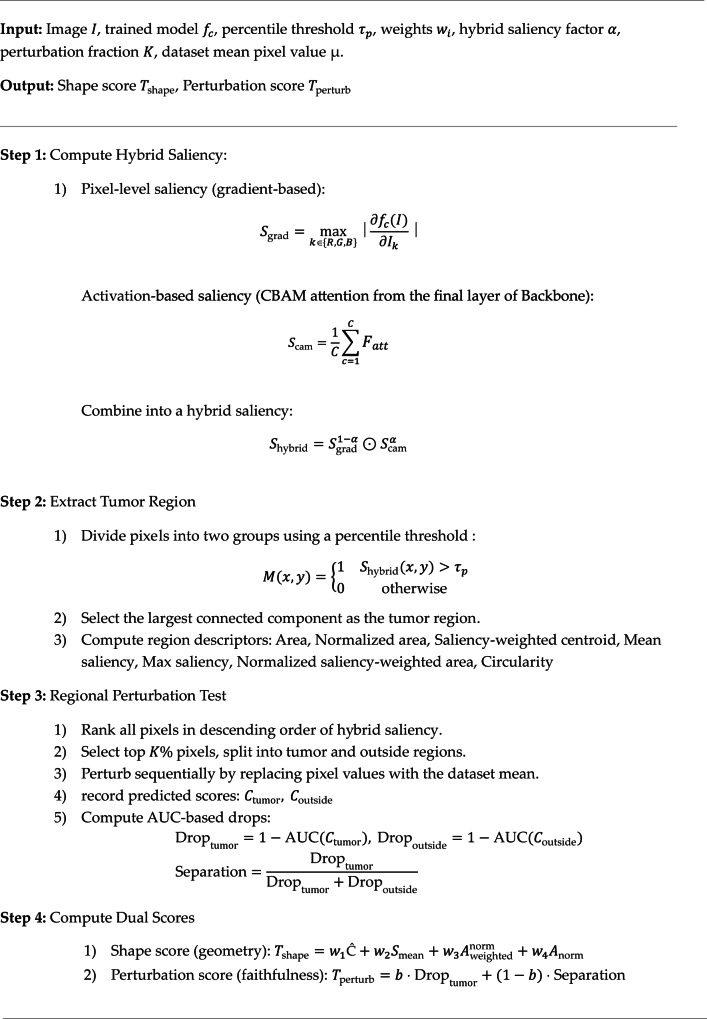
Algorithm: Dual-Score Regional XAI

## Results

Training and evaluation of the proposed framework were performed on a system equipped with two Nvidia Tesla T4 GPUs having 29GB RAM and 4 CPU cores. Deep Learning was implemented using Python (v3.12.12) and Pytorch (v2.8.0 + cu126) with supporting libraries such as Torchvision (v0.23.0 + cu126), NumPy(v2.0.2), Scikit-learn (v1.6.1), Pillow (v11.3.0), SciPy (v1.15.3) and Matplotlib (v3.10.0). This setup enabled faster training and evaluation with moderate batch sizes (batch_size = 32). To ensure reproducibility, fixed random seeds (random_seed(42)) were applied for all processes therefore all randomized operations could be replicated. All experiments were conducted on the same hardware setup, ensuring fair comparison.

### Dataset and preprocessing

**Fig. 5:**
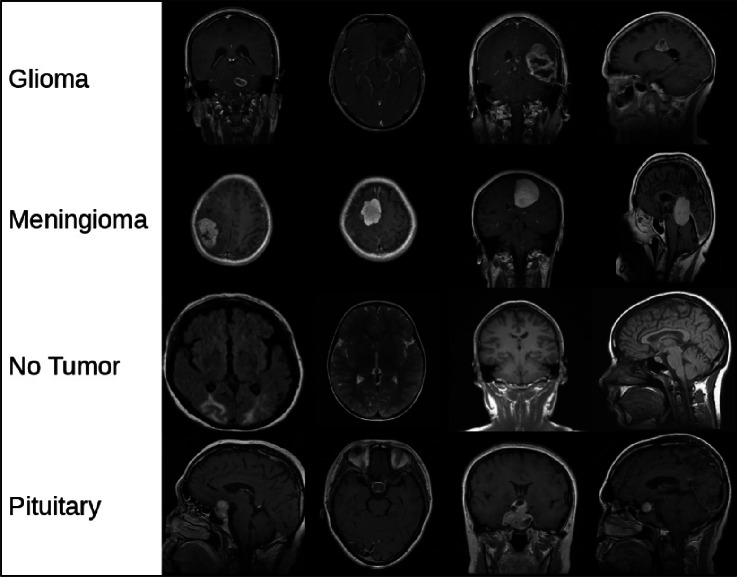
Representative MRI samples from the dataset. Each row shows the three anatomical views (axial, coronal, sagittal) for one tumor class. From top to bottom: glioma, meningioma, no tumor (healthy), and pituitary tumor. The dataset exhibits significant variability in tumor appearance, size, and contrast across different views and classes.

The Dataset used for all experiments is publicly available on Kaggle^37^. This dataset contains 7023 images of human brain MRI images which are classified into 4 classes: glioma, meningioma, no tumor and pituitary (Table 1). The training subset contains 5,712 images and the testing subset consists of 1,311 images. All images are stored in JPEG format. The MRI images are in Axial, Coronal and Sagittal views as shown in Fig. 5.

**Table 1:** Dataset used in this study.

Class	Training (Train + Validation)	Testing
Glioma	1,321	300
Meningioma	1,339	306
No Tumor	1,595	405
Pituitary	1,457	300
Total	**5**,**712**	**1**,**311**

For the proposed MLHnet model, 5- fold stratified cross validation was used to ensure reliable estimation of metrics. For each fold, 80 − 20 split was taken for the train and validation sets. After validation, the trained model for each fold was tested on the testing subset of 1311 images. The XAI experiments were performed on the testing subset only. The ablation study was performed using the training, validation and test split used in fold 3 which yielded the highest result for the proposed model.

All MRI images are converted to RGB format and resized to 224 by 224 pixels to ensure compatibility with CNN backbones. Pixel values were normalized to [0,1]. Data augmentation was applied online to the training set to improve model robustness, meaning that different transformed variants of the original images were generated dynamically during training. The augmentation pipeline included colour jittering with brightness variation up to 0.6, contrast up to 1.0, saturation up to 0.3, and hue up to 0.1. In addition, images were randomly rotated within ± 90 degrees and horizontally and vertically flipped with a probability of 0.5.

### Training and testing setup

The model was trained using 5-fold stratified cross validation for 50 epochs per fold. The cross-entropy loss function was used to optimize the multi-class objective. AdamW^38^ optimization was implemented to update the model parameters with a learning rate of 1 × 10⁻⁴. It combines Adaptive Momentum learning with weight regularisation (weight_decay = 0.001), which helps in model generalisation. An early stopping strategy was applied where the training loop was terminated when the validation loss did not improve for a defined number of epochs (15 epochs). This technique helps to cutoff the training before it overfits the training subset.

After training and validation, the trained model was evaluated on the testing subset. Test metrics were computed for each fold, and final reported results were obtained by averaging the metrics, providing a reliable estimate of model performance. The metrics used to evaluate were accuracy, precision, recall, F1-score, area under the ROC curve, and confusion matrices. Macro-averaging was used to obtain multi-class results. Let the confusion matrix have True Positive (TP), True Negative (TN), False Positive (FP), False Negative (FN). Metrics were calculated by the following formulas:

34 $$\:\mathrm{Accuracy}=\frac{TP+TN}{TP+TN+FP+FN}$$

35 $$\:\mathrm{Precision}=\frac{TP}{TP+FP}$$

36 $$\:\mathrm{Recall}=\frac{TP}{TP+FN}$$

37 $$\:\mathrm{F1-score}=2\times\:\frac{\mathrm{Precision}\times\:\mathrm{Recall}}{\mathrm{Precision}+\mathrm{Recall}}$$

38 $$\:\mathrm{Specificity}=\frac{TN}{TN+FP}$$

39 $$\:\mathrm{AUC}={\int\:}_{0}^{1}TPR\:\left(FPR\right)\:d\left(FPR\right)$$

where:

$\:\mathrm{TPR}=\frac{TP}{TP+FN}$ (True Positive Rate) $\:\mathrm{FPR}=\frac{FP}{FP+TN}$ (False Positive Rate).

After all 5 folds were completed, these result metrics were compared and averaged to evaluate model consistency.

### Explainability setup

The Explainability experiment was conducted on the test set, excluding the no tumor class, to focus on regions relevant to tumor detection. The trained weights of the best performing model were used. The Perturb value is the mean pixel value of the validation subset. The shape weights were calculated empirically from the validation set. The validation subset is used to determine dataset related constants to reduce model bias. Perturbation curves were recorded for each image and then averaged across classes to evaluate the model’s reaction to perturbing salient regions. Class-wise Box plots were calculated for the shape score to view inter-class variation. The XAI hyperparameters used are given in Table 2.

**Table 2:** Hyperparameters used in the Dual-Score Regional XAI framework.

Parameter	Default Value	Description
α (Saliency Map constant)	0.7	Balance the weight between gradient-based and activation-based saliency maps
Threshold_percentile (*τ*_*p*_)	90	Percentile cutoff for extracting the tumor region from the saliency map
Perturb_value	[0.15, 0.15, 0.15]	Mean RGB pixel intensity of the validation set used for perturbation
Top_K_percent	1%	Fraction of most salient pixels analyzed in perturbation test
shape_weights	[0.0002, 0.0569, 0.7818, 0.1611]	Weights for circularity, mean saliency, weighted area, and normalized area in Shape Score
b (Tumor Drop constant)	0.6	Balance the parameter between Tumor Drop and Separation in Perturb Score

### 5-fold cross-validation

The training and validation metrics are given in Table 3. The model performs consistently well with an average accuracy of 99.13% across folds. The model does not appear to be overfitted, as the average validation accuracy is 98.92%, which is very close to the training performance. From the Table 3, we can also infer that the model of fold 3 is highly robust, wherein the validation accuracy even exceeds the training accuracy. Across all folds, the training subset achieved average precision, recall, F1-score, and specificity values above 99%, while the corresponding validation metrics remained consistently high at approximately 98.9%.

**Table 3:** Train and validation performance across 5-fold cross-validation.

Fold	Dataset	Accuracy (%)	Precision (%)	Recall (%)	F1-score (%)	Specificity (%)
1	Train	99.10	99.08	99.07	99.07	99.69
	Val	98.78	98.72	98.82	98.77	99.58
2	Train	99.30	99.28	99.28	99.28	99.76
	Val	99.13	99.12	99.08	99.10	99.64
3	Train	99.26	99.26	99.24	99.25	99.74
	Val	99.74	99.72	99.73	99.73	99.89
4	Train	98.82	98.80	98.78	98.79	99.54
	Val	98.34	98.31	98.24	98.27	99.32
5	Train	99.17	99.14	99.14	99.14	99.67
	Val	98.60	98.58	98.59	98.59	99.46

**Fig. 6:**
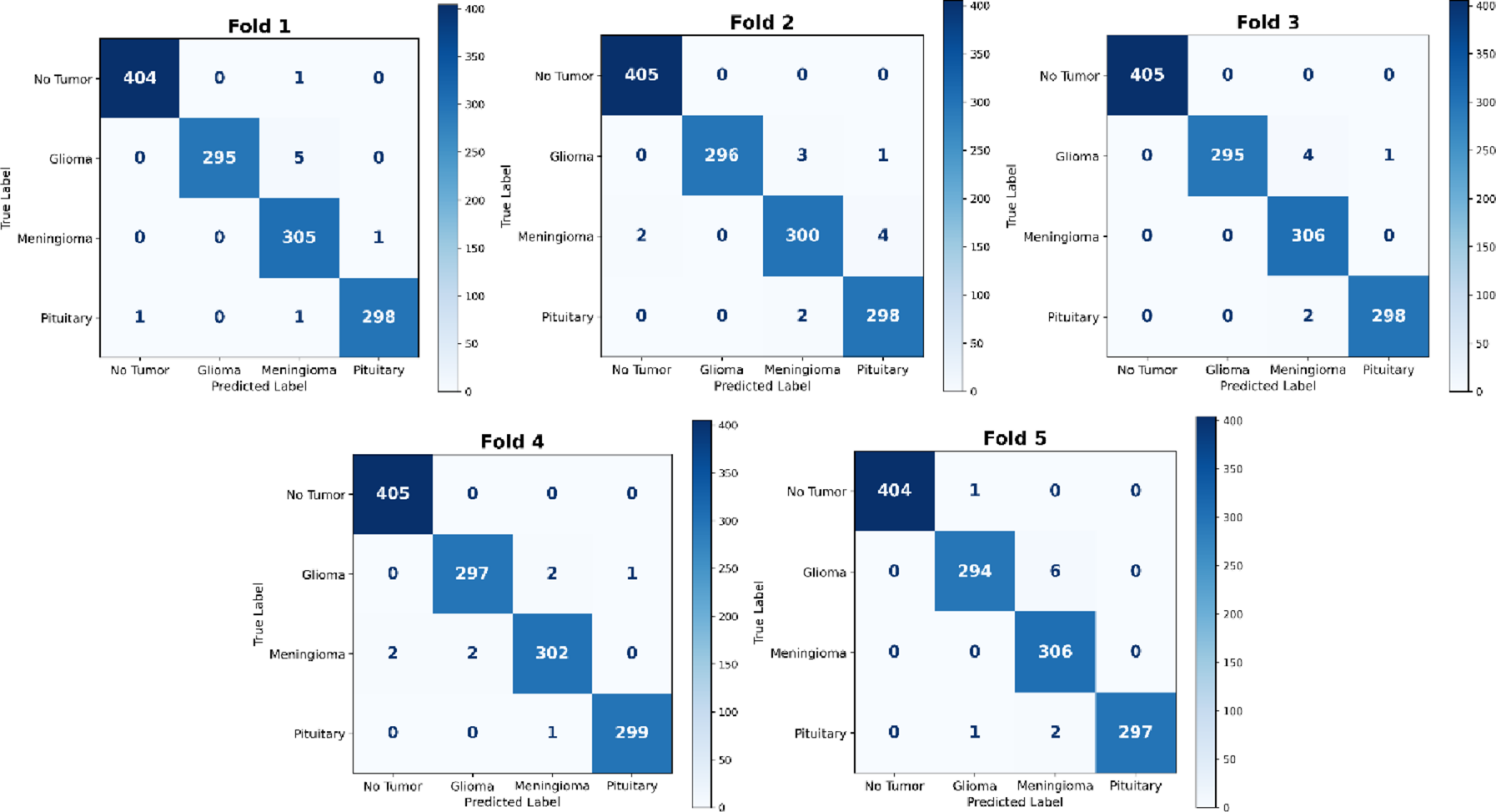
Confusion matrices for all five folds on test set (1311 images).

Table 4 shows performance metrics on the test set for all the folds. With this data we can judge which model has learnt the best and has good generalisation capability. All folds display similarly high scores exceeding 99% across most metrics, with low variance, highlighting the model’s consistently strong performance. The Confusion Matrices of each fold can be visualised in Fig. 6. It is observed that healthy class images have a near perfect prediction record. The Glioma class is often misclassified as the Meningioma class because of their similar appearance. The Pituitary class has a fairly accurate prediction record, though it was confused with meningioma in a few cases.

**Table 4:** Test results for each fold.

Fold	Accuracy (%)	Precision (%)	Recall (%)	F1-score (%)	Specificity (%)	AUC (%)
1	99.31	99.29	99.27	99.28	99.77	99.97
2	99.08	99.05	99.01	99.03	99.70	99.98
3	99.47	99.44	99.42	99.42	99.83	99.93
4	99.39	99.38	99.34	99.36	99.80	99.99
5	99.24	99.19	99.19	99.19	99.75	99.97
Average	**99.30 ± 0.14**	**99.27 ± 0.15**	**99.25 ± 0.15**	**99.26 ± 0.15**	**99.77 ± 0.04**	**99.97 ± 0.02**

Among the 5 folds, fold 3 has a superior validation and test performance with an accuracy of 99.74% and 99.47%, respectively. Therefore, the weights from fold 3 were used in the proposed XAI framework.

### Dual-score Regional XAI

**Fig. 7:**
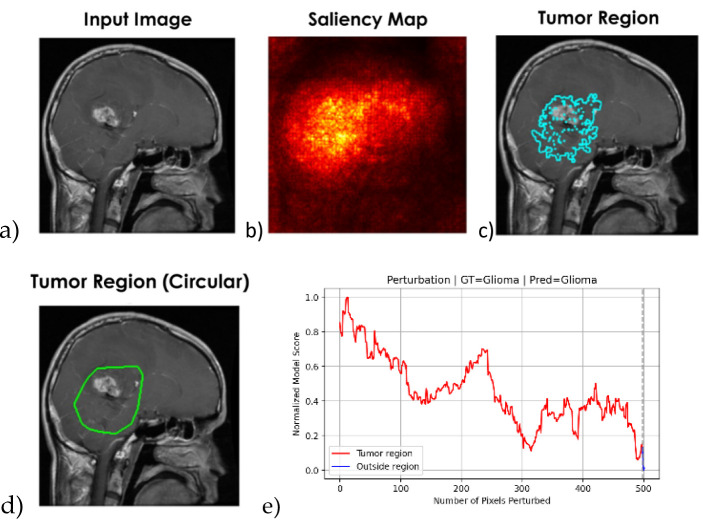
Step-by-step visualization of the Dual-Score Regional XAI pipeline for a glioma sample. (a) Original MRI input image; (b) Hybrid saliency map combining gradient and activation information; (c) Raw tumor region extracted via percentile thresholding (τₚ = 90); (d) Refined tumor region after morphological post-processing; (e) Regional perturbation curves showing the drop in model confidence as salient pixels within (red) and outside (blue) the tumor region is progressively masked.

The purpose of Dual Score in the proposed XAI is to access and quantify the faithfulness of the explanations as well as how different the explanations for each class are. As shown in Fig. 7, the input image is first processed to generate a hybrid saliency map. This saliency map is then used to localize a raw tumor region, which is further refined through post‑processing to obtain the final tumor region. A region‑wise perturbation test is subsequently applied to evaluate the impact of the localized region on the model’s prediction.

The metrics obtained from the proposed XAI method are shown in Table 5. Model confidence represents the certainty associated with the predicted class. The proposed Dual Score consists of two components: the Shape Score and the Perturb Score.

The Shape Score quantifies class‑wise differences in explanations based on the shape and structural characteristics of the localized tumor region. It includes circularity, which reflects how closely the region resembles a circular shape; mean saliency, defined as the average saliency intensity within the tumor region; weighted area, which combines region size with saliency importance, and normalized area. A low standard deviation across these shape metrics indicates consistent and distinguishable class‑specific explanations.

The Perturb Score measures the model’s sensitivity to perturbations applied within the localized tumor region. Tumor Drop captures the reduction in prediction confidence when the identified tumor region is perturbed, providing a quantitative measure of explanation faithfulness.

**Table 5:** Class-wise statistics of Dual-Score Regional XAI metrics on the test set (excluding no tumor class, *N* = 906 images).

True Label	Confidence	Shape Score	Perturb Score	Tumor Drop	Separation	Area	Norm Area	Circularity	Mean Saliency	Weighted Area
Glioma	0.994 ± 0.039	0.253 ± 0.043	0.446 ± 0.100	0.504 ± 0.123	0.359 ± 0.105	3692.65 ± 507.71	0.074 ± 0.010	0.074 ± 0.032	0.489 ± 0.056	1790.126 ± 234.016
Meningioma	0.998 ± 0.015	0.333 ± 0.040	0.479 ± 0.142	0.564 ± 0.183	0.352 ± 0.081	4127.49 ± 305.50	0.082 ± 0.006	0.127 ± 0.040	0.400 ± 0.053	1646.400 ± 203.017
Pituitary	0.998 ± 0.022	0.144 ± 0.059	0.442 ± 0.123	0.493 ± 0.157	0.365 ± 0.088	2208.21 ± 976.46	0.044 ± 0.019	0.057 ± 0.028	0.567 ± 0.057	1213.468 ± 479.315

**Fig. 8:**
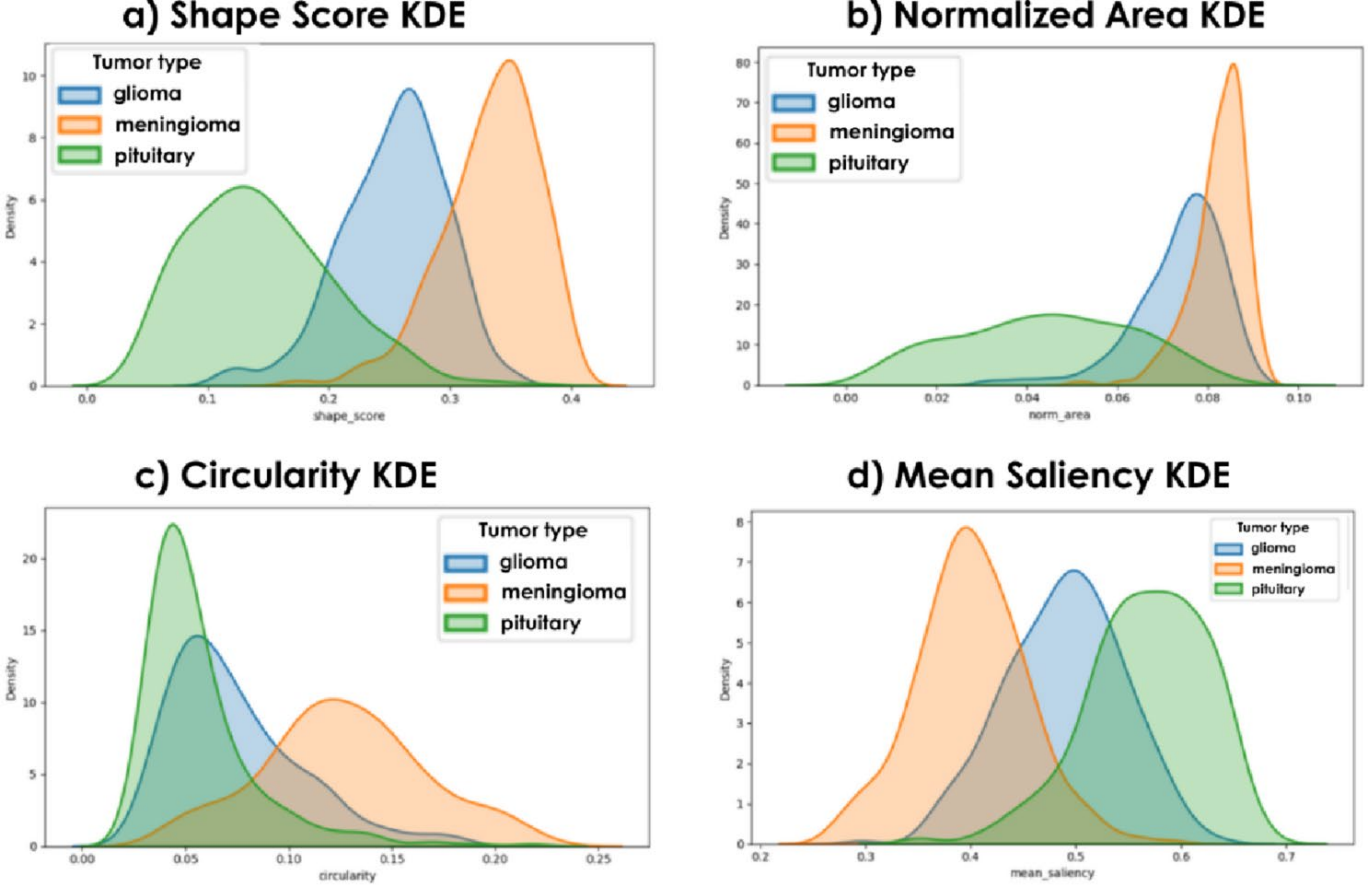
Class-wise Kernel Density Estimate plots of (a) Shape Score, (b) Normalised Area, (c) Circularity, (d) Mean Saliency.

The Kernel Density Estimate (KDE) plots in Fig. 8 demonstrate the class-agnostic scoring mechanism.

Meningioma exhibits the highest mean confidence. It also has the largest area with a more circular shape than other classes. A higher tumor drop score indicates that perturbing the tumor region leads to a significant drop in model confidence. Pituitary tumors show high confidence while showing small tumor regions based on the shape score. A high mean saliency indicates how the model focuses on a small specific salient region. The model finds pituitary regions the hardest to localize, as indicated by the lowest perturb score among the classes. Glioma has lower confidence with more irregular tumor boundaries. It has a moderate score across all metrics, except for a high weighted area, which shows emphasis of the model on the tumor region.

**Fig. 9:**
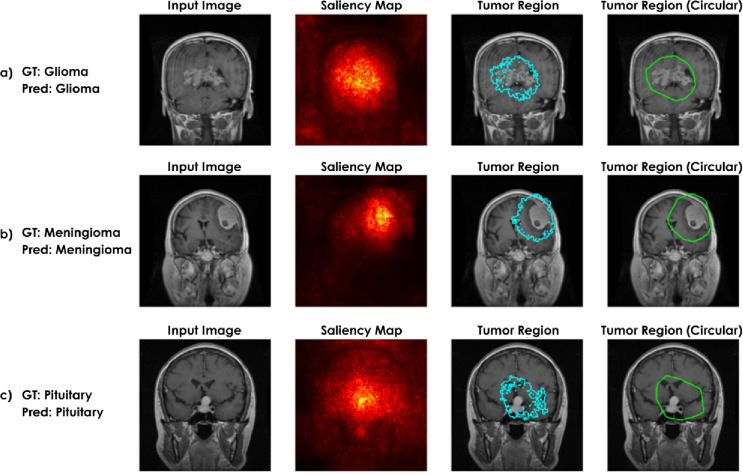
Examples of correctly localized tumor regions validated by a radiologist. Each row represents a different tumor type (top to bottom: glioma, meningioma, pituitary). The columns are the input MRI image, the saliency map generated by Hybrid Saliency, the raw tumor region correctly discovered and the smoothened tumor region for better visualisation.

**Fig. 10:**
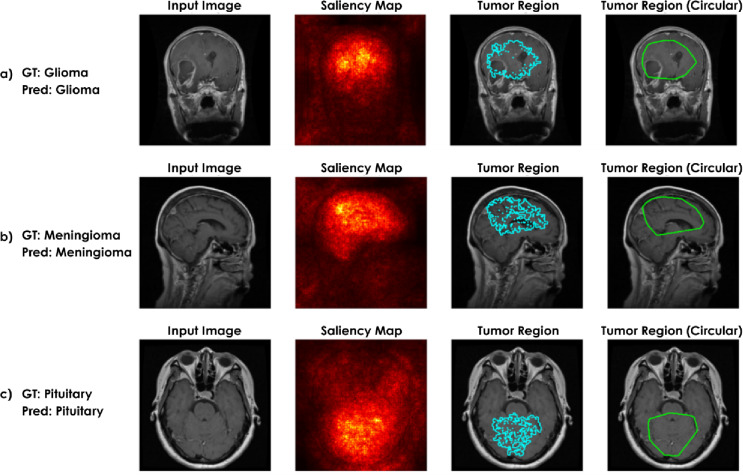
Examples of incorrectly localized tumor regions. The XAI framework struggles when saliency is diffusely distributed across large areas or exhibits multiple local maxima.

The clinical validity of the generated explanations was assessed by a radiologist’s evaluation. Representative examples of correctly localized tumor regions are shown in Fig. 9, demonstrating the framework’s ability to identify clinically relevant areas with high fidelity, particularly for meningiomas and gliomas. However, failure cases illustrated in Fig. 10 reveal limitations when saliency is diffusely distributed or contains multiple competing maxima, particularly in pituitary tumor images. These cases account for the lower explanation correctness rate (74.33%) observed for pituitary tumors compared to other classes.

Table 6 presents the expert evaluation of the XAI-generated tumor localization results. In the present study, the explanation maps were reviewed by a single experienced board-certified radiologist, who assessed whether the highlighted regions corresponded to clinically relevant tumor areas. A total of 906 test images, excluding the no-tumor class, were examined. An explanation was considered correct when the identified region corresponded to the clinically relevant tumor location. Overall, the proposed XAI framework achieved an explanation correctness of 87.64%. Among all tumor classes, meningioma achieved the highest explanation correctness (99.35%), whereas pituitary tumor showed the lowest correctness (74.33%), indicating greater difficulty in localizing this category.

**Table 6:** Expert validation of XAI-generated tumor localizations by a board-certified radiologist. A total of 906 test images (excluding the no tumor class) were evaluated. An explanation is considered correct if the identified region corresponds to the clinically relevant tumor location.

Tumor Type	Correctly Explained	Incorrectly Explained	Total	% Correct
Glioma	267	33	300	89.00%
Meningioma	304	2	306	99.35%
Pituitary	223	77	300	74.33%
Overall	**794**	**112**	**906**	**87.64%**

### Ablation Study

The proposed architecture is designed to capture complex brain tumor characteristics with correct context-based decision making. While each component of the proposed model may not add much to the performance itself, together they synergize with each other improving overall performance and reliability as shown in Table 7. The Efficientnet-B0 serves as a strong baseline; however, it lacks any contextual understanding necessary to scale up to complex cases. To address this, a transformer module is added in series. When used without well‑structured tokens or large‑scale training data, the transformer alone leads to a performance drop. Similarly, the inclusion of relative positional bias, while beneficial for spatial awareness in principle, introduces additional noise in the absence of robust token representations, further degrading performance. To address the problem of poor token representation, we integrated Multi-Level Feature Extraction, resulting in a substantial improvement in accuracy. This indicates the benefit of combining low-level texture information with high-level semantic information. It was seen that GAP used in the MLFE component, treated all spatial locations uniformly. GeM was added to concentrate pooling only on highly salient features using a learnable parameter. This component alone did not improve performance, as it tended to prioritize visually dominant regions rather than clinically relevant ones. Finally, CBAM is integrated to explicitly guide the GeM mechanism toward tumor-relevant features, effectively making clinically important regions the most salient. With CBAM, the model learns to prioritize diagnostically meaningful features rather than visually dominant patterns. This final addition completes the proposed MLHnet architecture, in which all components synergistically collaborate to achieve an accuracy of 99.47%, demonstrating that each module contributes uniquely to the overall performance.

**Table 7:** Results of ablation study.

Efficient-NetB0	Transformer	RPB	MLFE	GeM	CBAM	Accuracy (%)	Precision (%)	Recall (%)	F1-score (%)
✓	✗	✗	✗	✗	✗	99.01	98.98	98.97	98.97
✓	✓	✗	✗	✗	✗	98.93	98.93	98.88	98.90
✓	✓	✓	✗	✗	✗	98.86	98.79	98.83	98.80
✓	✓	✓	✓	✗	✗	99.24	99.23	99.17	99.20
✓	✓	✓	✓	✓	✗	99.08	99.05	99.03	99.04
✓	✓	✓	✓	✓	✓	99.47	99.44	99.42	99.42

RPB: Relative Position Bias, MLFE: Multi Level Feature Extraction.

To further justify the adopted fusion design, we additionally compared the proposed concatenation strategy with attention-based^39^, bilinear^40^, and weighted fusion^41^ alternatives under identical architectural and training settings. The comparison results are reported in Table 8.

**Table 8:** Comparison of fusion strategies.

Fusion Strategy	Accuracy (%)	Precision (%)	Recall (%)	F1-score (%)
Concatenation	**99.47**	**99.44**	**99.42**	**99.42**
Attention-based^39^	99.24	99.24	99.17	99.21
Bilinear Fusion^40^	99.31	99.30	99.28	99.29
Weighted Fusion^41^	99.31	99.28	99.27	99.28

### Comparison with state-of-the-art

Table 9 summarizes the performance of the proposed model alongside recent state-of-the-art (SOTA) models evaluated on the publicly available Brain MRI Kaggle dataset. Although all studies were conducted on the MRI brain datasets, differences in preprocessing, augmentation, data partitioning, and training protocols may affect strict comparability of the reported results. Within this context, the proposed framework demonstrates strong performance, achieving an accuracy of 99.47% with consistently high precision, recall, and F1-score, while also providing an interpretable XAI component for model explanation. In addition, the model is lightweight, with a size of 21.27 MB, 5.31 million learnable parameters, and a computational cost of 1.61 GFLOPs using float32 precision. These characteristics indicate its potential suitability for efficient deployment in resource-constrained settings.

**Table 9:** Comparison with state-of-the-art methods on various brain MRI datasets available on Kaggle.

State of the Art Models	Year	XAI	Accuracy (%)	Precision (%)	Recall (%)	F1-score (%)
A-GRU [17]	2024	✗	99.32	100	99.12	99.01
VGG16-Resnet50-CDBN fusion [^42^]	2024	✗	98.98	97.95	97.82	97.87
BTDN [18]	2025	✗	95.33	94.89	94.78	94.91
EFFResNet-ViT [27]	2025	✓	99.31	99.26	99.25	99.26
PDSCNN-RRELM [32]	2025	✓	99.5	100	100	100
BRAIN-META [25]	2026	✓	97.1	97	96.92	96.94
ShallowMRI with Self Attention Learning [28]	2026	✗	98.24	98.17	98.1	98.13
Proposed Model	**2026**	**✓**	**99.47**	**99.44**	**99.42**	**99.42**

## Discussion

### Impact of the Dual-Score Regional XAI

**Fig. 11:**
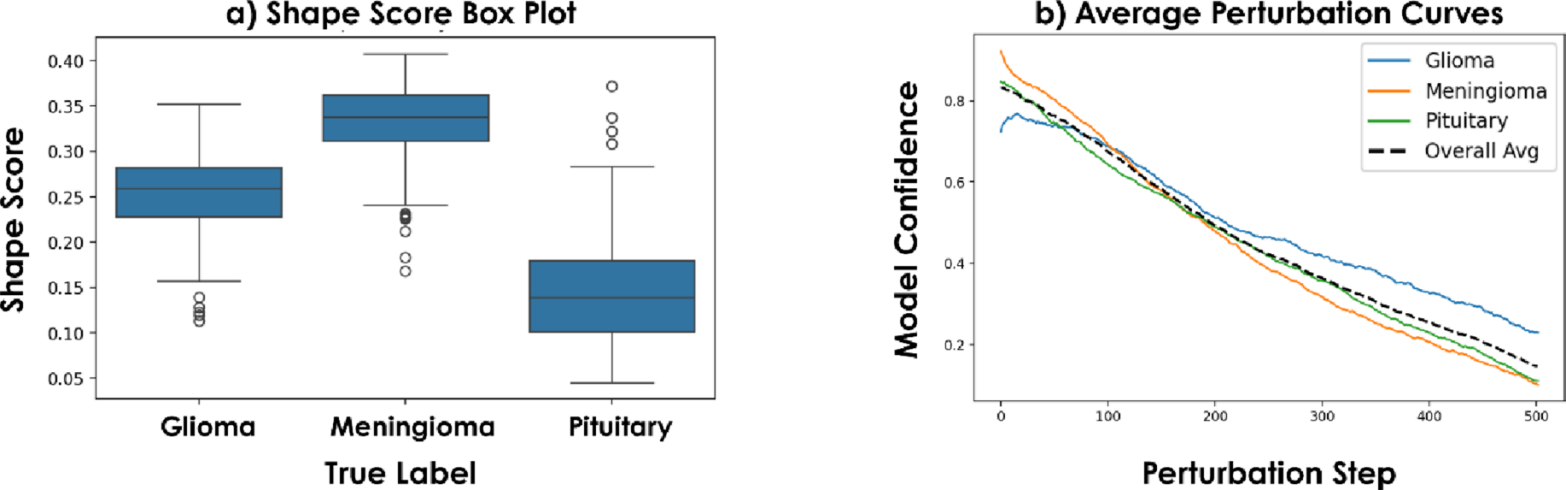
Quantitative analysis of XAI explanations across tumor classes. (a) Box plot of Shape Scores showing class-specific geometric characteristics (b) Average perturbation curves demonstrating the drop in model confidence as the top 1% most salient pixels are progressively masked. The consistent downward trend across all classes validated that the model relies on the identified tumor regions for prediction.

The results of Dual-Score Regional XAI for the entire test set are aggregated and averaged, as illustrated by Fig. 11. The Shape Score Box Plot (Fig. 11 (a)) shows the proposed XAI to differentiate tumor classes based on shape score. The interquartile ranges of all classes are apart from eachother, whereas the whiskers and outliers overlap with eachother. This highlights the complexity of the brain tumor classification problem. It is seen that Meningioma is often higher than other classes of tumor. The interquartile ranges of glioma and meningioma are close to each other, suggesting similarity in the shape characteristics of these two tumor classes. The Pituitary tumor class is smaller than the rest of the classes, as suggested by its low position on the plot. The Average Perturbation Curve by class (Fig. 11 (b)) shows a steady decrease across all classes, indicating a drop in model confidence when the pixels within the generated tumor region are perturbed. The proposed XAI method was conducted exclusively on tumor-positive samples. It does not provide interpretability for healthy classes or false negative predictions.

To verify the competency of Hybrid Saliency used for Dual-Score Regional XAI, a comparative study is done, taking inspiration from Kakogeorgiou and Karantzalos ’s paper on XAI^43^. While experimenting, two metrics were calculated MoRF AUC and Computation Time. The perturbation value was taken as 0.15 and the perturbation was done pixelwise for 500 iterations. Final MoRF curves were normalised for better readability. Lower MoRF AUC values indicate better identification of truly salient regions by the XAI method.

**Fig. 12:**
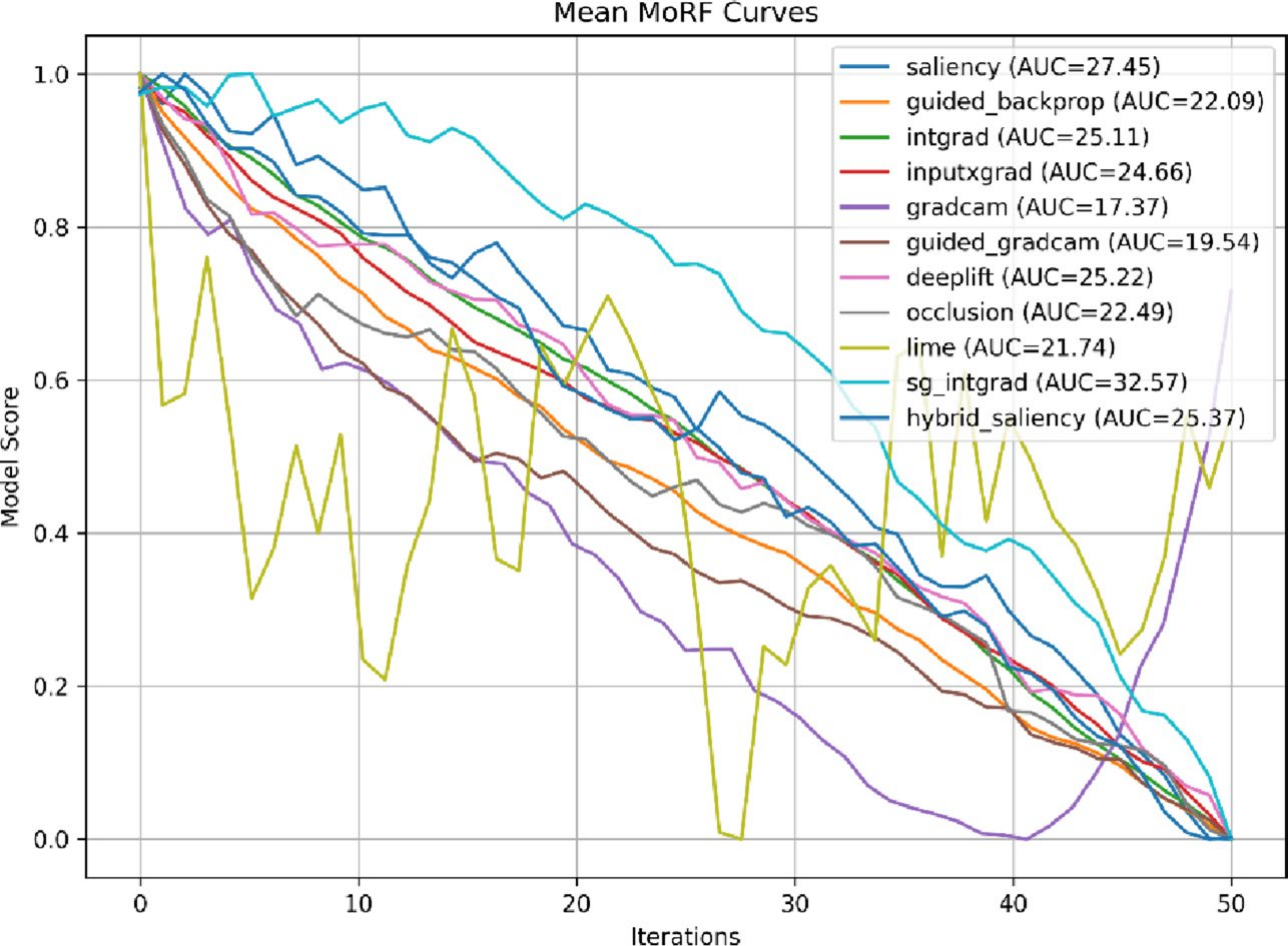
Comparison of XAI methods using Most Relevant First (MoRF) perturbation analysis. The curves show normalized model confidence as the most salient pixels are progressively masked.

Table 10 displays the performance of XAI methods, and Fig. 12 illustrates the MoRF curves calculated. Saliency^44^ is the baseline model as it is simple with acceptable scores across both metrics. GradCAM^9^ achieves the lowest MoRF AUC with the fastest computation time indicating its popularity. However, as the perturbation increases, the model starts to detect artifacts, artificially increasing the model score as seen in the plot. Guided GradCAM^9^ also performs competitively but at a higher computation time. LIME^11^ is highly susceptible to artifacts. Occlusion^10^ and SmoothGrad-Integrated Gradients^45^ have very high computational time with poor trade-off, as MoRF AUC is subpar as well. Deeplift^46^ has a high MoRF AUC and the lowest computation time; however, it depends on baseline selection, causing attribution bias. Guided-Backpropagation^47^, InputxGradient^46^ and Integrated Gradients^48^ have a favourable tradeoff between MoRF AUC and Computation time where Integrated Gradients has more computation time. To make the proposed XAI framework, high-performance and low complexity are achieved using Hybrid Saliency, which gives a mean MoRF AUC of 25.3667 and a mean computation time of 0.9070 s.

**Table 10:** Quantitative comparison of XAI methods using Most Relevant First (MoRF) perturbation analysis.

XAI Method	Mean MoRF AUC (↓)	Mean Computation Time (s) (↓)
Saliency	27.4530	0.9362
Input x Gradient	24.6613	0.9910
Integrated Gradients	25.1077	3.3984
Guided Backpropagation	22.0926	0.9313
Gradient-weighted Class Activation Mapping (Grad-CAM)	17.3712	0.8707
Guided Grad-CAM	19.5394	0.9743
Deep Learning Important FeaTures (DeepLIFT)	25.2155	0.7949
Occlusion	22.4891	17.2085
Local Interpretable Model-agnostic Explanations (LIME)	21.7401	17.1666
SmoothGrad Integrated Gradients	32.5691	23.6708
Hybrid_Saliency	**25.3667**	**0.9070**

### Advantages of the proposed system

- The proposed model achieves strong performance on a widely used public brain MRI dataset and compares favorably with recent reported methods.
- The proposed architecture benefits from rich hierarchical feature representations, tumor localization and global-local contextual learning.
- The proposed explainability framework provides interpretable region-based visualizations and quantitative scores that support analysis of model behavior.
- The proposed model maintains a balanced trade-off performance and computational efficiency, enabling potential real-time clinical deployment.

### Limitations and Future Directions

The proposed model was trained and evaluated on a single publicly available dataset. Although this dataset is widely used, reliance on a single source may limit the generalizability of the findings across institutions, scanners, acquisition protocols, patient populations, and real-world clinical distributions. Therefore, external validation on independent cohorts is necessary before making broader claims regarding robustness, clinical reliability, or practical applicability.

In addition, although the proposed XAI framework provided encouraging results, its radiologist-based evaluation indicates that there remains room for improvement in the clinical fidelity of the generated explanations. The current explainability pipeline also relies on empirically defined thresholds, hyperparameters, and scoring criteria, which should be further validated across different datasets and clinical settings. In particular, the framework may fail to precisely localize the tumor region when saliency is broadly distributed over large areas, even if local maxima are still present within the true abnormal region. Future work will therefore investigate improved hyperparameter tuning and more effective post-processing strategies to better isolate tumor-relevant regions. Beyond this, the proposed architecture could be extended to process entire MRI volumes rather than individual 2D images, while the XAI module could be adapted to generate and analyze 3D saliency maps. The framework may also be explored in other medical imaging domains, including ultrasound and retinal imaging, to assess its broader applicability.

## Conclusion

In this study, we propose the MLHnet, a CNN–Transformer hybrid architecture that leverages both mid‑level and final‑level feature extraction to capture fine‑grained texture details and high‑level semantic information. The proposed architecture achieves state‑of‑the‑art performance, achieving an accuracy of 99.47% on a publicly available brain MRI Kaggle dataset. To address the black‑box nature of deep learning models, we also introduce the Dual‑Score Regional XAI framework, which localizes tumor-relevant regions and quantifies explanation faithfulness. The resulting system is lightweight, computationally efficient, and clinically interpretable.

The robustness and effectiveness of the proposed system are validated through extensive experiments, including 5‑fold cross‑validation, radiologist evaluation, ablation studies, and comparative analyses with existing methods. Nevertheless, since the study was conducted on a single public dataset, further validation on independent external datasets and real-world clinical cohorts is required before drawing broader conclusions regarding generalizability, robustness, or clinical applicability.

## Data Availability

The datasets generated and/or analysed during the current study are available in the Kaggle repository, https://www.kaggle.com/datasets/masoudnickparvar/brain-tumor-mri-dataset (Version 1).
